# Cranial Radiation Therapy as Salvage in the Treatment of Relapsed Primary CNS Lymphoma

**DOI:** 10.3390/curroncol29110644

**Published:** 2022-10-28

**Authors:** Matthew E. Volpini, Jiheon Song, Rajiv Samant, David MacDonald, Vimoj J. Nair

**Affiliations:** 1 Division of Radiation Oncology, The Ottawa Hospital, Ottawa, ON K1H 8L6, Canada; 2 Division of Hematology, The Ottawa Hospital, Ottawa, ON K1H 8L6, Canada

**Keywords:** primary CNS lymphoma, cranial radiation therapy, whole brain radiation therapy, salvage radiation therapy

## Abstract

Primary central nervous system lymphoma (PCNSL) is a rare malignancy. Standard of care is upfront high-dose methotrexate (HD-MTX) chemotherapy, while cranial radiation is more commonly used in the salvage setting. In this retrospective study, we aimed to investigate the safety and efficacy of salvage cranial radiation in PCNSL. PCNSL patients who received upfront HD-MTX chemotherapy and salvage cranial radiation after treatment failure between 1995 and 2018 were selected. Radiological response to cranial radiation was assessed as per Response Assessment in Neuro-Oncology Criteria. Twenty one patients were selected (median age 59.9 years), with median follow-up of 19.9 months. Fourteen patients (66.7%) received a boost to the gross tumour volume (GTV). Four patients (19.0%) sustained grade ≥2 treatment-related neurotoxicity post-completion of cranial radiation. Of the 19 patients who had requisite MRI with gadolinium imaging available for Response Assessment in Neuro-Oncology (RANO) criteria assessment, 47.4% achieved complete response, 47.4% achieved partial response, and 5.3% of patients exhibited stable disease. Higher dose to the whole brain (>30 Gy) was associated with higher rate of complete response (63.6%) than lower dose (≤30 Gy, 37.5%), while boost dose to the gross disease was also associated with higher rate of complete response (61.5%) compared with no boost dose (33.3%). Median overall survival was 20.0 months. PCNSL patients who relapsed following upfront chemotherapy showed a high rate of response to salvage cranial radiation, especially in those receiving greater than 30 Gy to the whole brain and boost to gross disease.

## 1. Introduction

Primary central nervous system lymphoma (PCNSL) accounts for 3% of primary central nervous system (CNS) tumours and is a challenging malignancy to treat [1]. Early work in the treatment of PCNSL investigated the efficacy of upfront whole brain radiation therapy (WBRT). While response rates were generally favourable, median survival rates were modest—on the order of 12–18 months, with 5-year overall survival (OS) of just 4% [2]. Subsequent work investigated the use of high-dose methotrexate (HD-MTX) chemotherapy and adjuvant WBRT, which resulted in improved survival outcomes compared with the historical controls [3,4,5,6,7]. Studies reported a median OS ranging from 25–60 months [3,4,5,6,7]. However, adjuvant WBRT was also associated with higher rates of neurotoxicity, particularly in older patients [3,5,6,8,9,10]. In an attempt to limit neurotoxicity, the use of WBRT in the salvage setting was investigated and it was found that salvage WBRT compared with adjuvant WBRT resulted in similar survival outcomes and lower rates of neurotoxicity [11,12]. As such, cranial radiation has been increasingly employed as salvage therapy in patients with incomplete response to chemotherapy, disease progression or recurrence after chemotherapy [11,12,13]. Without the use of salvage radiation therapy, patient prognosis is quite poor, on the order of short months in prior retrospective series [14,15]. Cranial radiation therapy remains an option, however, due to the rarity of disease the role of salvage cranial radiation therapy remains unclear and requires further study. We aimed to investigate the efficacy and safety of salvage cranial radiation therapy in PCNSL among patients treated at our center. 

## 2. Materials and Methods

Institution Review Board approval was obtained prior to commencement of the study. A retrospective study was conducted on patients with pathologic diagnosis of PCNSL, who were found to have treatment failure following chemotherapy, and were offered cranial radiation therapy (WBRT with or without boost) between 1995–2018. 

Upfront HD-MTX chemotherapy was administered according to hospital protocol. In general, induction cycles were administered every 14 days until complete response followed by consolidation treatments every 14 days for two cycles. Serial MR imaging was used to assess response. Following completion of HD-MTX chemotherapy, eligible patients were considered for consolidative autologous stem cell transplant. 

The main endpoints were response to cranial radiation, overall survival, and neurotoxicity. Response to cranial radiation was determined by considering best response on magnetic resonance imaging (MRI) with gadolinium contrast administration as per Response Assessment in Neuro-Oncology (RANO) Criteria [16].CR was achieved if no gadolinium enhancement could be appreciated, partial response (PR) was achieved if there was a >50% reduction in cross-sectional area of the enhancing lesion(s), and progression was defined as >25% cross-sectional area growth in the enhancing lesion(s). All other radiographic results were interpreted as stable disease burden [14]. Unfortunately, owing to lack of consistent documentation regarding performance status and steroid requirement, these factors could not be utilized in our assessment of response. Survival was measured from the completion of cranial radiation. To identify patients with delayed neurologic deficits related to cranial radiation, semi-annual follow-up reports were reviewed to identify subjective (concentration difficulties, short-term memory deficits, and increased fatigue) as well as objective (gait instability, bowel/bladder dysfunction) evidence of subcortical symptoms. These clinical findings were then cross-referenced with appropriately timed neuroimaging (where available) to evaluate disease progression. Baseline patient characteristics are listed in Table 1.

Statistical analysis was performed using IBM^®^ SPSS Statistics 27.0 (Armonk, NY, USA). Survival was analyzed with the Kaplan–Meier methods. OS was defined as time interval between completion of radiation therapy and death from any cause, and progression-free survival (PFS) was defined as time interval between completion of radiation therapy and either disease progression or death from any cause, whichever occurred first.

## 3. Results

**Figure 1:**
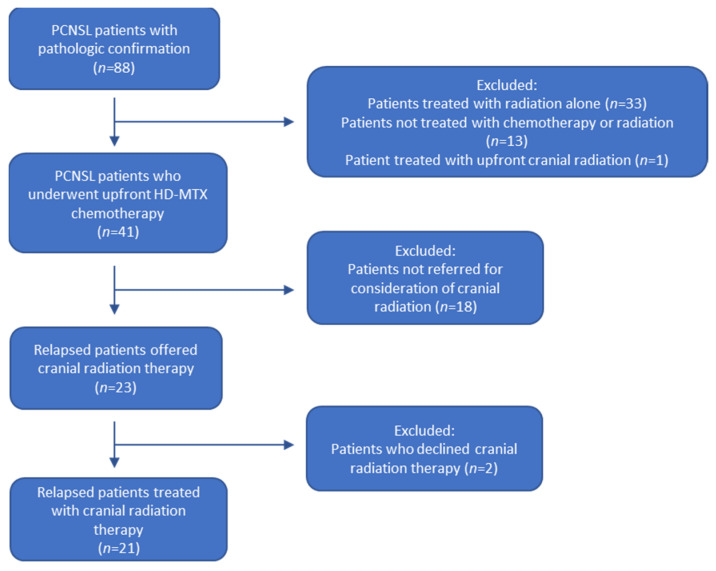
Flow diagram for patient selection. PCNSL: primary central nervous system lymphoma. HD-MTX: High-dose methotrexate.

A total of 220 patients with CNS lymphoma were identified in our database. Eighty-eight of these patients had a pathologically confirmed diagnosis of primary CNS lymphoma, who were assessed by both Hematologic Oncology and Radiation Oncology. Of these, 47 patients were excluded as they did not receive upfront HD-MTX chemotherapy (33 patients were treated with radiation therapy alone, 13 were not treated with chemotherapy or radiation therapy, and 1 patient was treated with upfront cranial radiation therapy). Forty-one patients were treated with upfront HD-MTX chemotherapy. Eighteen of these patients were excluded as they were not referred for consideration of salvage radiation therapy (including patients in long-term remission, patients who relapsed but were treated with second-line systemic therapy, and patients who relapsed but were not referred for consideration of radiation therapy). This left a total of 23 consecutive patients with pathologic diagnosis of PCNSL who had relapsed or progressed after chemotherapy who were offered salvage cranial radiation therapy. Of these 23 patients, 21 chose to proceed with radiation therapy (Figure 1). All patients had pathological diagnosis of diffuse large B-cell lymphoma (DLBCL) from either biopsy or surgical resection. At time of diagnosis, all patients underwent staging investigations with computed tomography (CT) scans of the body, which confirmed no evidence of extra-CNS disease. Six patients were confirmed seronegative for human immunodeficiency virus (HIV), while the remaining 17 patients were not tested, and the status remained unknown. Two patients (8.7%) achieved an initial CR following 6 cycles of chemotherapy, while an additional 7 patients (30.4%) achieved an initial PR following a median of 5 cycles of chemotherapy, for a total response rate of 39.1%. For responders, the median time to failure as measured from the first dose of chemotherapy to progression or recurrence was 5.1 months (range 1.9 months to 47.5 months). Six patients (26.1%) had no response to chemotherapy, while 8 patients (34.8%) progressed while receiving chemotherapy. No patients received an autologous stem cell transplant, though this would now be considered standard of care. Chemotherapy was ultimately discontinued due to progression or recurrence in all patients. Please see Table 1 for additional patient characteristics.

### 3.1. Prescribing Practices and Initiation of Cranial Radiation Therapy

Cranial radiation therapy consisted of WBRT with or without a boost. WBRT was planned with opposed lateral beams encompassing the cranium and extending inferiorly to the level of the C2 vertebra, encompassing the entire cranial meninges. Implementation of a boost was accomplished with the addition of conformal beams, and MRI fusion to aid target delineation. The average age at time of cranial radiation was 59.9 years (range 37.3 to 72.3 years). The median time from PCNSL diagnosis to first fraction of cranial radiation was 4.5 months (range 1.7 to 47.8 months). Patient performance status was not consistently documented at time of radiation oncology consultation. Of the 21 patients treated with cranial radiation, 14 received a boost to the gross tumour volume (GTV) via involved field radiation therapy. For patients who received a boost, the median whole brain dose was 34.5 Gy (range 30 Gy to 45 Gy), and the median localized boost dose to the GTV was 10 Gy (range 5 Gy to 24 Gy). For patients who did not receive a boost, the median whole brain dose was 30Gy (range 20 Gy to 45 Gy). For all patients, the median fraction size was 2 Gy, as was the most common fraction size (range 1.70 Gy to 4 Gy). 

In our cohort, 60% of patients ≤60 received a WBRT >30 Gy compared to 54.5% of patients over age 60. Patients over the age of 60 made up 50% of patients who received a WBRT dose >30 Gy, and 55.5% of patients who received a WBRT dose ≤30 Gy. Furthermore, 50% of patients ≤60 received a boost, while 81.8% of patients >60 received a boost. Patients over age 60 made up 64.3% of patients receiving a boost, and 40% of patients not receiving a boost.

Considering the number of lesions, 16.7% of patients with solitary brain lesions received a WBRT dose >30 Gy, compared with 73.3% of patients with multiple foci of disease. Furthermore, 91.7% of patients who received a WBRT dose >30 Gy had multifocal disease compared with just 44.4% of patients who received a WBRT dose <30 Gy, while 50% of patients with solitary brain lesions and 50% of patients with multiple lesions received a boost. 

With respect to maximum tumour size, 58.3% of patients with maximum tumour size ≤3 cm received a WBRT dose >30 Gy compared to 55.6% of patients with lesions >3 cm. In addition, patients with tumour size >3 cm made up 41.7% of patients who received a WBRT dose >30 Gy compared with 44.4% of patients with WBRT dose ≤30 Gy. Furthermore, 50% of patients with smaller index lesions received a boost to gross disease, whereas 88.9% of patients with larger index lesions received a boost. Furthermore, patients with larger lesions made up 57.1% of those who received a boost to gross disease, compared with just 14.2% of patients who did not receive a boost.

With respect to deep structure involvement, 41.7% of patients without deep structure involvement received a WBRT dose >30 Gy, compared with 77.8% of patients without deep structure involvement. In addition, 58.3% of patients who received WBRT dose >30 Gy had deep structure involvement, while 22.2% of patients who received WBRT ≤30 Gy had deep structure involvement. In addition, 50% of patients without deep structure involvement received a boost, compared with 88.9% of patients with deep structure involvement. Additionally, 57.1% of patients who received a boost to gross disease had deep structure involvement, while the rate of deep structure involvement among patients who did not receive a boost was just 14.3%.

All patients completed their courses of prescribed radiation and were maintained on oral corticosteroids for the duration of their radiation treatment, with a trial of taper shortly following completion of cranial radiation therapy, as per protocol.

### 3.2. Response to Cranial Radiation Therapy

**Figure 2:**
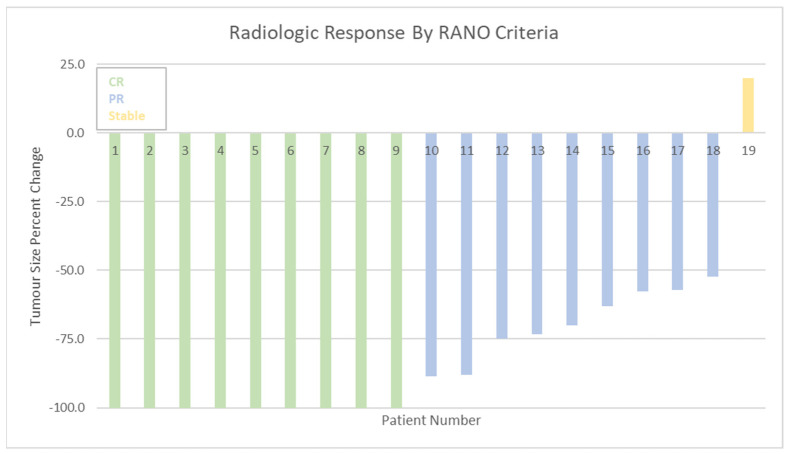
Response rates as per RANO criteria. RANO = Response Assessment in Neuro-Oncology. CR = complete response. PR = partial response. Stable = stable disease.

Of the 21 patients who completed cranial radiation therapy, 19 had the requisite pre- and post- treatment MRI with gadolinium contrast administration which formed the basis for RANO criteria assessment. Two other patients had pre-treatment MRI with gadolinium enhancement, but post-treatment imaging in the form of computed tomography (CT) head with contrast. For the 19 patients with requisite imaging, the best radiologic response was recorded for each patient following completion of cranial radiation therapy. Nine patients (47.4%) attained a CR, and an additional 9 patients (47.4%) attained a PR for a total response rate of 94.8% (Figure 2). One patient (5.3%) had stable disease post cranial radiation and no patients exhibited progression. Of the 10 patients who achieved PR or SD following cranial radiation therapy, 5 ultimately recurred (4 patients exhibited progression of their original lesion, 1 patient developed a new lesion), with median time from best radiologic response to recurrence of 2.9 months (range 1.5–14.5 months). No patients received additional radiation therapy. One patient went on to receive salvage systemic therapy in the form of cyclophosphamide, doxorubicin, vincristine and prednisolone, but was transferred to hospice and died 2.1 months following initiation of salvage therapy. Of the 9 patients who achieved CR following cranial radiation, 3 recurred, with median time from best radiologic response to recurrence of 21.6 months (range 11.2–67.2 months). Each of these patients died within one month of recurrence, and did not receive salvage therapy.

Of the 19 cranial radiation patients analyzed by RANO criteria, a total of 7 (36.8%) were prior responders to up-front chemotherapy (either CR, or PR). Of these 7 responders to chemotherapy, all 7 (100%) were found to be subsequent responders to cranial radiation therapy, with 5 patients (71.4%) achieving CR following cranial radiation. Of the 12 patients who were non-responders to chemotherapy, 11 (91.7%) responded to cranial radiation therapy, with 4 (36.4%) achieving CR following cranial radiation therapy. Of the 11 patients who received a WBRT dose >30 Gy, 10 (90.9%) were responders, with 3 (27.3%) achieving PR and 7 (63.6%) achieving a CR. One patient (9.1%) exhibited stable disease. All 8 patients who received a WBRT dose ≤30 Gy were found to be responders, with 5 (62.5%) achieving PR and 3 (37.5%) achieving CR. Of the 13 patients who received a boost to gross disease, 12 (92.3%) were responders, with 4 (30.1%) achieving PR, and 8 (61.5%) achieving CR. One patient (7.7%) had stable disease. All 6 patients who did not receive a boost were found to be responders, with 4 (66.7%) achieving PR and 2 (33.3%) achieving CR. 

### 3.3. Survival

**Figure 3:**
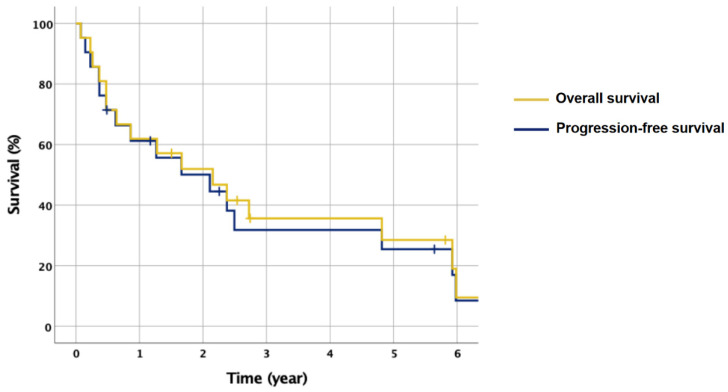
Kaplan–Meier survival curves for patients treated with cranial radiation therapy. Median overall survival (OS): 20.0 months (range 0.9 to 143.6 months). Median progression-free survival (PFS): 14.1 months (0.9 to 111.4 months).

At time of analysis, 16 of the 21 patients who underwent cranial radiation therapy had died. Of the deceased, 14 patients died from PCNSL, while one patient died from a stroke with brain imaging negative for recurrence 7 days prior to death, and another patient died of end-stage renal disease and no evidence of clinical relapse. Median OS from the completion of radiation therapy was 20.0 months (range 0.9 months to 143.6 months), while median progression-free survival (PFS) was 14.1 months (range 0.9 months to 111.4 months) Figure 3. 

**Figure 4:**
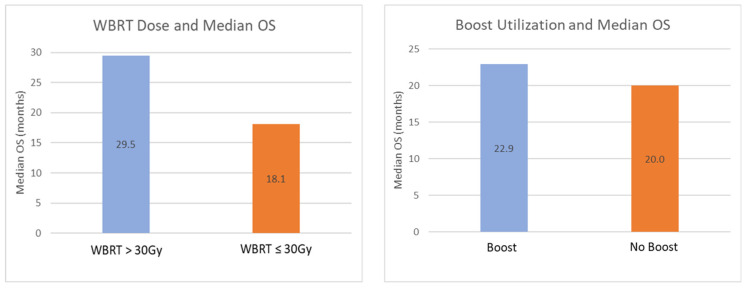
Median OS by WBRT dose (**Left**), and the implementation of a boost to gross disease (**Right**). WBRT dose >30 Gy was associated with marked increase in median OS = overall survival.

Median OS was 29.5 months (range 0.9 to 143.6 months) for 12 patients receiving WBRT >30 Gy, and 18.1 months (range 5.7 to 71.1 months) for 9 patients receiving WBRT ≤30 Gy. Median OS was 22.9 months (range 0.9 to 143.6 months) for 14 patients receiving boost, and 20.0 months (range 5.7 to 71.8 months) for 7 patients without a boost. Median OS for the 10 patients ≤60 years old was 24.2 months (range 2.7 to 143.6 months), compared to 15.3 months (range 0.9 to 69.8 months) among the 11 patients >60 years old (Figure 4). 

In our cohort, two patients who relapsed after up-front HD-MTX were offered salvage WBRT and declined. The first patient lived 0.6 months, and the second patient lived 1.2 months after relapse.

### 3.4. Radiation Toxicity

Four patients (19.0%) sustained confirmed or probable neurotoxicity secondary to radiation treatment, diagnosed at 14, 15, 18 and 59 months post-completion of cranial radiation therapy. The first confirmed case relates to a patient who presented 15 months post-WBRT with gait changes and cognitive decline indicative of subcortical pathology. The repeat MRI did not show any evidence of disease recurrence, but there was evidence of treatment-related white matter disease. The second definite case relates to a patient who similarly presented with gait instability and confusion nearly five years post-RT. Repeat MRI was negative for disease recurrence, but white matter changes consistent with radiation treatment were appreciated. The two cases of probable treatment-related neurotoxicity involve symptomatic patients with repeat imaging showing mixed evidence of disease progression and treatment-related white matter changes. Specifically, the first patient presented 18 months post-WBRT with cognitive decline. Repeat brain imaging revealed evidence of disease progression, but also white matter changes related to radiation treatment. The second patient similarly presented with fatigue and confusion 14 months post-WBRT, with imaging showing treatment-related white matter changes and disease progression.

All cases of neurotoxicity were observed in patients who received both a whole brain dose >30 Gy and a boost. Of patients ≤age 60, one patient (10%) sustained neurotoxicity, while 3 patients (27.3%) >age 60 sustained neurotoxicity. 

## 4. Discussion

Our results suggest that salvage cranial radiation therapy is an effective and relatively safe treatment option for patients with PCNSL who experience disease progression or relapse following upfront HD-MTX chemotherapy. The median OS measured from time of diagnosis was 30.3 months in our cohort, which is similar to previous trials where patients were treated with upfront combination HD-MTX plus WBRT, where OS ranged from 25–33 months [5,7,9,17]. This suggests that cranial radiation therapy can be used in the salvage setting without compromising overall survival benefit, and this is preferable given the increased rates of neurotoxicity observed when WBRT is used in the adjuvant setting [3,5,6,8,9,10]. Median OS from the start of WBRT was 20.0 months, which is also consistent with previously reported series [17,18]. In our cohort, two patients were offered WBRT and declined therapy opting instead for best supportive care. The first patient lived 0.6 months, and the second patient lived 1.2 months after relapse, which provides further support for the short natural history of patients with relapsed PCNSL who are treated with best supportive care alone [18,19]. Given the natural history of this disease in the absence of cranial radiation, it is reasonable to conclude that OS gains are indeed due to radiotherapy, especially given the excellent radiologic response rates exhibited post-cranial radiation therapy.

In our cohort, patients over 60 accounted for 55.5% of the patients receiving ≤30Gy and 50% of patients receiving >30 Gy. While these proportions are not dissimilar given our modest cohort size, we can not rule out the possibility that response or survival outcomes were affected by selection bias, as younger patients were more likely to receive higher WBRT dose. Interestingly, patients over 60 were more likely to be prescribed a boost compared to younger patients ≤60 (81.8% versus 50%, respectively), and patients over 60 made up the majority of the patients receiving a boost (64.3%) compared with just 40% of the patients not receiving a boost. Increasing age is a negative prognostic factor, and it is interesting to note that in our cohort, survival and response outcomes were slightly better among patients who received a boost, despite there being a higher proportion of older patients in this group [20,21].

In our cohort we found that patients with multifocal disease were more likely to receive a WBRT >30 Gy (73.3%) compared with patients with solitary lesions (16.7%). The rates of boost implementation was not found to differ based on lesion number, with 50% of patients with solitary lesions and 50% of patients with multiple lesions prescribed a boost. Prior studies have failed to demonstrate that the number of brain lesions in patients with PCNSL is an independent prognostic factor for survival or response [20,21,22]. That being said, we recognize that for our specific cohort of patients, we can not definitively state that our outcomes were not affected by the higher proportion of patients with multiple lesions represented in the WBRT >30 Gy treatment group.

Our data shows that 58.3% of patients with maximum tumour size ≤3 cm received WRBT >30 Gy compared to 55.6% of patients with maximum tumour size <3 cm. These rates are not markedly different given the small size of our cohort. Larger maximum tumour size was associated with increased rates of boost utilization, however, with 88.9% of patients with tumor size >3 cm receiving a boost, compared to 50% of patients with tumour size ≤3 cm. There is conflicting evidence in the literature regarding size of the primary tumour as a prognostic factor [21,23,24]. While no consensus exists regarding the nature of tumour size as a prognostic factor in PCNSL, we can not be certain that it did not influence outcomes in our particular study, given that the proportion of patients with tumours >3 cm made up 57.1% of those receiving a boost and 14.2% of those receiving no boost.

In our cohort, patients with deep structure involvement were more likely to receive a WBRT dose >30 Gy, compared with no deep structure involvement (77.8% versus 41.4%, respectively). Similarly, patients with deep structure involvement were more likely to receive a boost (88.9% versus 50%, respectively). Deep structure involvement is a known negative prognostic factor [21,25,26]. In our cohort, median OS and radiologic CR rates were found to be higher for patients who received a higher WBRT dose >30 Gy, and also for patients who received a boost. It is interesting to note that these outcomes were observed in our cohort even though patients with deep structure involvement made up 58.3% of patients receiving WBRT dose >30 Gy, and comparatively accounted for just 22.2% of patients who received WBRT dose ≤30 Gy. Similarly, patients with deep structure involvement accounted for 57.1% of patients who received a boost and comparatively accounted for just 14.3% of patients who did not receive a boost. Given that survival gains and improved radiologic CR were observed with higher dose and the implementation of a boost, despite the higher proportion of patients with deep structure involvement, it may be possible that the true benefit of higher WBRT dose or the implementation of a boost are underestimated in our cohort.

The overall cranial radiation therapy response rate in our cohort was robust at 94.8% (47.4% CR and 47.4% PR) which is consistent with previously reported retrospective series [17,18]. In our cohort, initial responders to up-front HD-MTX chemotherapy achieved a subsequent 71.4% CR rate following cranial radiation therapy, while non-responders to chemotherapy had a lower CR of 36.4%, which suggests that response status to upfront chemotherapy may predict higher response rates to cranial radiation therapy. One concept that may explain this trend is the cancer stem cell hypothesis which posits that cancer stem cells possess genetic adaptations which confer cross resistance to both radiation therapy and chemotherapy, including efficient DNA repair, resistance to apoptosis, and slowed cell cycle kinetics [27,28]. 

Our results suggest that higher WBRT dose and the implementation of a boost to gross disease may be associated with increased response rates. Patients receiving a WBRT dose >30 Gy had a CR rate of 63.6% compared to 37.5% among patients who received ≤30 Gy, while patients who received a GTV boost exhibited a CR rate of 61.5%, compared to 33.3% for patients who did not receive a boost. Similarly, our data suggests that increased WBRT dose was associated with improved OS, as patients who received a WBRT dose >30Gy had a median OS of 29.5 months, compared to median OS of 18.1 months for those receiving ≤30 Gy. Median OS was 22.9 months for patients who received a boost to gross disease, and 20.0 months for patients who did not receive a boost. 

There was also evidence that higher WBRT dose and the use of a boost were associated with increased rates of treatment-related neurotoxicity. In total, there were four confirmed or probable cases of radiation induced neurotoxicity diagnosed at 14, 15, 18, and 59 months post-completion of RT, all of whom received WBRT dose of at least 30 Gy and a boost to gross disease. There were no cases of neurotoxicity among patients receiving a WBRT dose ≤30 Gy, or in patients who did not receive a boost. Taken together, one interpretation of this result is that higher whole-brain dose and the use of a boost may be associated with a higher risk of neurotoxicity. However, patients receiving higher doses with boost may also be living long enough to sustain neurotoxicity secondary to their radiation treatment, as these patients also exhibited improved OS in our cohort. The observed neurotoxicity rate in our series is 19% is similar to a prior series where WBRT was used in the salvage setting, which reported a neurotoxicity rate of 15% [18]. When analyzed by age, our cohort exhibited a higher incidence of neurotoxicity in older patients [7,8,18] with 75% of the observed cases of neurotoxicity occurring in patients over age 60. Therefore, we caution the use of concomitant WBRT dose >30 Gy and boost in this age group. Another point to consider when selecting a prescription is the close association between progression and death in this cohort, as demonstrated by the closely approximated PFS and OS curves. Physicians and patients need to weigh the risk of radiation related toxicity against the goal of delaying progression, since progression is symptomatically worse and associated with death.

As our study is limited by the lack of a control group, we can not definitely state that cranial radiation therapy was responsible for the observed improvement in OS advantage. This study is also limited by modest cohort size, lack of consistent documentation of patient performance status and steroid requirement throughout treatment duration and follow up, and the single center nature of this study. In our cohort, we found that patients with multifocal disease were more likely to be prescribed WBRT dose >30 Gy, while patients with maximum tumour size >3cm were more likely to receive both WBRT dose >30 Gy and a boost to gross disease. The impacts of lesion number and size as independent prognostic factors have not yet been established. As such, our report is limited by the potential impact that these prescribing disparities may have had on our outcomes (survival, radiologic response).

## 5. Conclusions

Our results provide additional evidence that cranial radiation is an effective and relatively safe treatment for PCNSL among patients who relapse following HD-MTX chemotherapy. Practitioners need to employ a balanced approach when deciding on treatment, as higher WBRT dose and the implementation of a boost were associated with improved response rates and survival outcomes, but were also associated with increased rates of neurotoxicity, particularly in the elderly. Continued work should focus on validating these results with larger cohorts, ideally across multiple centers.

## Figures and Tables

**Table 1:** Patient characteristics.

Number of Patients	21
Gender, number (%) -Male -Female	10 (47.6%) 11 (52.4%)
Age >60 years, number (%)	11 (52.4%)
Tumour type, number (%)	DLBCL, 21 (100%)
Initial systemic therapy	HD-MTX chemotherapy, all patients
Number of HD-MTX chemotherapy cycles, median (range)	5 cycles (1 to 6 cycles)
Time from diagnosis to cranial radiation therapy, median (range)	4.5 months (1.7 to 47.8 months)
Time from completion of HD-MTX chemotherapy to cranial radiation therapy, median (range)	1.5 months (0.5 to 44.6 months)
Patients with WBRT dose >30 Gy, number (%)	12 (57.1%)
Patients who received a boost to gross disease, number (%)	14 (66.7%)

## Data Availability

The data presented in this study are available on request from the corresponding author.
